# Inhibition of *Period* Gene Expression Causes Repression of Cell Cycle Progression and Cell Growth in the *Bombyx mori* Cells

**DOI:** 10.3389/fphys.2019.00537

**Published:** 2019-05-03

**Authors:** Jian-Feng Qiu, Xue Li, Wen-Zhao Cui, Xiao-Fei Liu, Hui Tao, Kun Yang, Tai-Ming Dai, Yang-Hu Sima, Shi-Qing Xu

**Affiliations:** ^1^School of Biology and Basic Medical Sciences, Medical College, Soochow University, Suzhou, China; ^2^Institute of Agricultural Biotechnology and Ecology (IABE), Soochow University, Suzhou, China

**Keywords:** *Period* gene, cell cycle, apoptosis, autophagy, ovarian cell line, *Bombyx mori*

## Abstract

Circadian clock system disorders can lead to uncontrolled cell proliferation, but the molecular mechanism remains unknown. We used a *Bombyx mori* animal model of single *Period* gene (*BmPer*) expression to investigate this mechanism. A slow growing developmental cell model (Per-KD) was isolated from a *B. mori* ovarian cell line (BmN) by continuous knock down of *BmPer* expression. The effects of *BmPer* expression knockdown (Per-KD) on cell proliferation and apoptosis were opposite to those of *m/hPer1* and *m/hPer2* in mammals. The knockdown of *BmPer* expression led to cell cycle deceleration with shrinking of the BmN cell nucleus, and significant inhibition of nuclear DNA synthesis and cell proliferation. It also promoted autophagy via the lysosomal pathway, and accelerated apoptosis via the caspase pathway.

## Introduction

The circadian clock system and cell cycle are closely related (Shostak et al., 2016), and cell cycle progression occurs at a specific time in the circadian rhythm (Scheving et al., 1983; Bjarnason and Jordan, 2000; Garcia et al., 2001; Smaaland et al., 2002). Some key proteins in cell cycle, such as Cyclin D1, Cyclin B1, Cyclin E, Cyclin A, P53, WEE1, C-MYC, P21, Mdm2, ATM, ATR and Gadd45, exhibit circadian rhythm-dependent expression (Pines, 1999; Delaunay and Laudet, 2002; Duffield, 2003; Sato et al., 2003). Regulation of the cell cycle by the circadian clock system has been observed in mouse hepatic cells (Matsuo et al., 2003) and mouse embryonic fibroblasts (MEFs) NIH3T3 (Bieler et al., 2014; Feillet et al., 2014), respectively. Altered expression of clock genes has been seen in cells with abnormal cell cycle kinetics or in rapidly dividing cancer cells (Fu and Kettner, 2013; Cadenas et al., 2014).

Circadian clock disorders or impairment have been reported to lead to abnormal cell proliferation and promotion of tumor growth in mouse models (Filipski et al., 2002, 2004; EI Cheikh et al., 2014). Knockout of the circadian clock genes *mCry1* and *mCry2* has been shown to accelerate the cell cycle in MEFs (Destici et al., 2011). Circadian clock disorders can directly inhibit the cell cycle, and the apoptosis of breast cancer cells (Blakeman et al., 2016). The results of some studies are inconsistent or contradictory. For example, knockout of the circadian clock gene *mClk* was reported to arrest the cell cycle and promote apoptosis in embryonic stem cells (Lu et al., 2016). We previously reported that knocking down the expression of the silkworm circadian clock *Period* gene (*BmPer*) inhibited the cell proliferation by repressing glycometabolism in *Bombyx mori* ovarian (BmN) cells (Tao et al., 2017). The mutual regulation of the circadian clock and cell cycle generates conflicting cellular signals and indicate that further analysis of the mechanism of circadian clock regulation of cell proliferation is necessary.

*Period* (*Per*) is a core component of the circadian clock transcriptional regulatory network (Dunlap, 1999; Takahashi et al., 2008). In mammals, mPER1 regulates multiple cell cycle proteins (Gery et al., 2006; Yang et al., 2009). mPER1 and mPER2 proteins have been shown to inhibit breast cancer *in vivo* by inducing cancer cell apoptosis (Fu et al., 2002; Gery et al., 2006; Blakeman et al., 2016). However, mammalian *mPer* has multiple subtypes with distinct temporal and spatial expression of functional protein products (Shearman et al., 2000; Bae et al., 2001; Cermakian et al., 2001; Zheng et al., 2001).

In this study, an animal model with a single *BmPer* gene product was selected to investigate the effect of Per-KD on the cell cycle and avoid the interaction of multiple *Per* expression products. There have been no previous reports of cell cycle changes after simultaneous knockdown or knockout of all *mPer* genes. A slow growing developmental model expressing a single *BmPer* gene that was continually knocked down in BmN cells (Per-KD) was used in this study. The BmN cells were free of endocrine influences. We compared cell proliferation and programmed cell death (PCD) and investigated the regulatory mechanisms in mutant and wild-type BmN cells.

## Materials and Methods

### Cell Preparation

**FIGURE 1:**
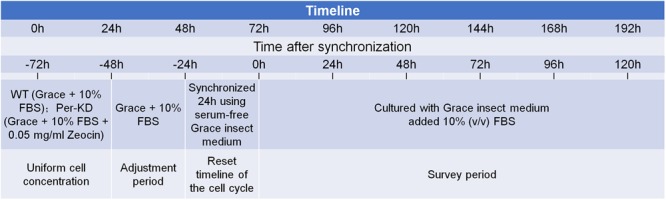
Study timeline and cell pretreatment.

A wild-type (WT) *B. mori* ovary cell line (BmN) and a mutant line with stable interference of the *BmPer* gene (Per-KD) (Tao et al., 2017), were maintained in our laboratory and cultured in Grace insect medium (11605094, GIBCO, United States) with 10% (v/v) fetal bovine serum (FBS) (04-121-1A; Biological Industries, United States) at 26°C in the dark. The medium for culture of Per-KD cells included 0.05 mg/mL Zeocin (R25001, Invitrogen, United States). As shown in Figure 1, cell lines were synchronized by 24 h culture in serum-free Grace insect medium. The medium was then replaced with Grace insect medium with 10% FBS (v/v). The cells were counted and adjusted to the desired concentration. The time at which the synchronization process ended was recorded as time 0 h after synchronization.

### Cell Proliferation Assay

After synchronization, the rate of cell division was determined at 0, 24, 48, 72, 96, and 120 h of growth in Grace insect medium with 10% (v/v) FBS with a methyl thiazolyl tetrazolium (MTT) assay (C0009, Beyotime, China). The cells (100 μL, 1 × 10^5^ cells/mL) were incubated for 4 h in 96-well plates at 26°C in the dark and additional 4 h at 37°C in the dark after adding 100 μL formazan. The absorbance at 570 nm was measured with an Eon microplate reader (BioTek, VT, United States). The measurement was repeated in five culture wells.

### Staining Methods

Synchronized BmN cells (1000 μL, 1.5 × 10^5^ cells/mL) were cultured in Grace insect medium with 10% (v/v) FBS. The cells were stained with using Click-iT^TM^ EdU Alexa Fluor^TM^ 488 Imaging Kits (C10337, Invitrogen, United States) following the manufacturer’s instructions (Salic and Mitchison, 2008; Ning et al., 2013), diamidino-phenyl-indole (DAPI; C1006, Beyotime, China) and TdT-mediated dUTP nick end labeling (TUNEL; 11684795910, Roche, Switzerland) as previously described (Liu et al., 2014; Li et al., 2017), monodansylcadaverine (MDC; G0170, Solarbio, China) as described by Biederbick et al. (1995), and Lyso-Tracker Red (C1046, Beyotime, China) as described by Yan et al. (2016). Immunohistochemical staining was performed using an anti-human cleaved-caspase-3 primary antibody (1:200, 9661s, CST, United States) and an Alexa Fluor 594-conjugated goat anti-mouse IgG (H+L) secondary antibody (1:300, AS054, ABclonal, China) as described by Ji et al. (2013).

### Flow Cytometry

Synchronized BmN cells (1000 μL,1 × 10^6^ cells/mL) were transferred to Eppendorf tubes containing Grace insect medium with 10% (v/v) FBS. Cell cycle and apoptosis assays were conducted simultaneously at 0, 24, 48, 72, 96, and 120 h. Cells were harvested by low speed centrifugation (4°C, 1000 rpm for 10 min), washed twice in precooled phosphate buffered saline (PBS; SH30256.01, HyClone, United States), resuspended in 1 mL PBS, and then fixed overnight at 4°C after adding 0.5 mL precooled 70% ethanol. Centrifugation and washing were repeated, and the cells were resuspended in 500 μL PBS. For flow cytometry, 5 μL RNase A and 5 μL 1 mg/mL propidium iodide (PI) were added to 100 μL of cell suspension and placed in direct sunlight for 30 min at 4°C before adding 400 μL PBS. The cell cycle assay was performed with an FC500 series flow cytometer (Beckman Coulter, Atlanta, GA, United States). Apoptosis was assayed with an annexin V-FITC/PI kit (AD10, Dojindo, Japan) following the manufacturer’s instructions.

### Real-Time Polymerase Chain Reaction (qRT-PCR)

The mRNA expression of marker genes was assayed by qRT-PCR using the primers shown in Supplementary Table S1 as described by Tao et al. (2017). Total RNA was extracted from WT and Per-KD BmN cells using RNAiso Plus kits (9109, TaKaRa, China), and DNA in the total RNA samples was digested using RNase-free DNase I (2270A, TaKaRa, China). cDNA was synthesized using PrimeScript RT reagent kits (RR037A, TaKaRa, China) following the manufacturer’s instructions, RT-qPCR was performed with Premix Ex Taq (RR420A, TaKaRa, China) using an ABI StepOne Plus (Life technologies, Marsiling Industrial Estate, Singapore) and the fluorescent dye SYBR in a total reaction volume of 20 μL. The reaction conditions were 95°C preinitiation heating for 30 s followed by 40 cycles at 95°C for 5 s and 60°C for 30 s. Melting curve analysis was used to confirm the amplification of specific products. The *BmRp49* gene was used to normalize the qRT-PCR results and a standard curve was used to determine the expression levels of the samples. All assays were performed in triplicate.

### Caspase-3 Activity Assay

WT and Per-KD cells (1.96 × 10^6^ cells) were cultured several days in Grace insect medium with 10% (v/v) FBS. Cells were collected by centrifugation (4°C, 1000 g, 10 min) at 24, 72, and 120 h after synchronization and then resuspended in 150 μL RIPA lysis buffer (P0013B, Beyotime, China) with 1% phenylmethanesulfonyl fluoride (PMSF; ST506, Beyotime, China). The suspended cells were lysed with an ultrasonic disruptor (Q700; Qsonica, Newtown, CT, United States) at 300 W, pulse on 3 s, pulse off 10 s, for 40 cycles. The lysate was centrifuged at 4°C at 12 000 rpm for 10 min. Caspase-3 activity in the supernatant was assayed with a colorimetric assay (BC3830; Solarbio, China) following the kit manufacturer’s instructions. The reaction system included 70 μL buffer solution, 25 μL sample and 5 μL acetyl-Asp-Glu-Val-Asp p-nitroanilide (Ac-DEVD-pNA). Buffer solution was used as the negative control. The assay was conducted for 2.5 h at 37°C in the dark. The absorbance at 405 nm was read with an Eon microplate reader (BioTek, VT, United States). Caspase-3 activity was estimated by comparison to a standard curve.

## Results

### Inhibition of *BmPer* Expression Causes Inhibition of the Cell Cycle

**FIGURE 2:**
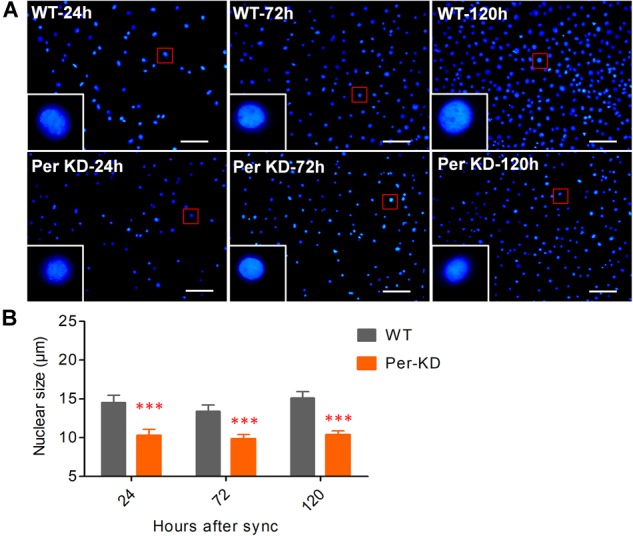
*BmPer* gene knockdown changes nuclear size. Cell lines were synchronized for 24 h in serum-free Grace insect medium. The nuclei were stained after 24, 72, and 120 h of culture in medium with 10% (v/v) FBS. **(A)** Cell nuclei were stained with 4’-6-diamidino-2-phenylindole (DAPI). Bar = 100 μm. **(B)** Nuclear size. ^∗∗∗^*p* < 0.001 (*n* = 3 plates with size measurement of 120 nuclei). WT, wild-type BmN cells; Per-KD, *BmPer* knockdown BmN cells.

We previously achieved continuous knockdown of *BmPer* gene expression in BmN cells and generated a Per-KD *B*. *mori* model with developmental disorders. The qPCR and western blotting results showed that *BmPer* gene transcription in Per-KD cells was downregulated by about 40% compared with WT cells and BmPER protein expression was significantly reduced by about 80%. *BmPer* gene knockdown thus significantly reduced both gene and protein expression of *Per* (Tao et al., 2017). In this study, DAPI staining of synchronized cell lines after culture for 24–120 h (Figure 2A) revealed that Per-KD nuclei were significantly smaller than WT nuclei (*p* < 0.001, Figure 2B). The difference in the number of DAPI-stained nuclei revealed that the proliferation rate of Per-KD cells was significantly lower than that of WT cells and that the difference increased with time (*p* < 0.001, Supplementary Figure S1).

**FIGURE 3:**
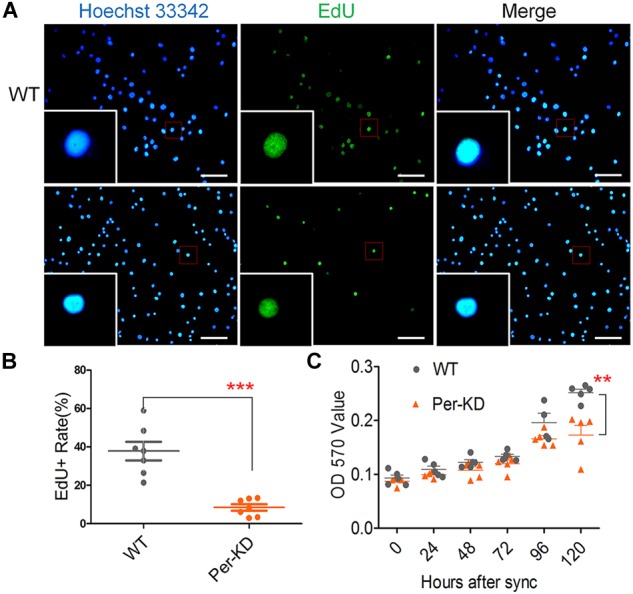
Changes in the proliferation of Per-KD cells following inhibition of nuclear DNA synthesis. Cells were synchronized for 24 h in serum-free Grace insect medium. The medium was replaced by Grace insect medium with 10% (v/v) FBS. After 8 h incubation with EdU **(A)** nuclei with green fluorescence indicate DNA synthesis. All nuclei show Blue Hoechst 33342 staining. Bar = 100 μm. **(B)** EdU-positive staining percentage (*n* = 7). **(C)** After synchronization and culture in Grace insect medium with 10% (v/v) FBS, the rate of cell division was determined with an MTT assay (*n* = 5). ^∗∗^*p* < 0.01, ^∗∗∗^*p* < 0.001.

*BmPer* knockdown affects the proliferation of BmN cells by inhibiting nuclear DNA synthesis. The effect of knockdown of *BmPer* expression on BmN cell proliferation was assayed by the amount of nuclear EdU incorporation over 8h. The difference in the percentage of EdU-positive cells indicated that the proliferation rate was significantly lower in Per-KD than in WT cells (*p* < 0.001, Figure 3A,B). Cell proliferation at 0–120 h after synchronization was also assayed by MTT. Compared with WT cells (Figure 3C), Per-KD cell proliferation began to slow at 72 h, reaching and became significantly different 120 h (*p* < 0.01).

### Cell Cycle Slowing in Per-KD Cells

**FIGURE 4:**
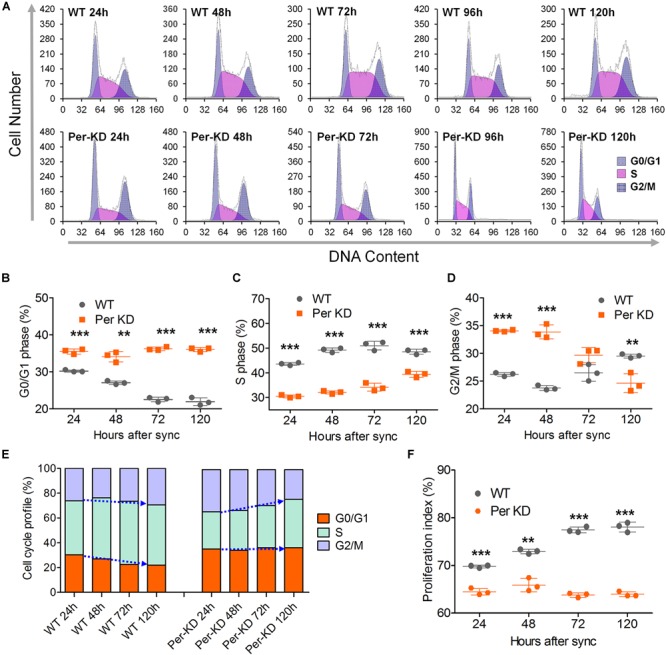
Cell cycle changes induced by *BmPer* gene knockdown. WT and *BmPer* knockdown BmN cells (Per-KD) were assayed 24, 48, 72, and 120 h after synchronization. **(A)** PI-FCM (Propidium Iodide-flow cytometry) results. **(B–D)** Percentages of cells in G0/G1 phase, S phase and G_2_/M phase. **(E)** Cell cycle profile. **(F)** Proliferation index = (S+G_2_/M)/(G_0_/G_1_+S+G_2_/M) × 100%. ^∗∗^*p* < 0.01, ^∗∗∗^*p* < 0.001 (*n* = 3).

The knockdown of *BmPer* expression resulted in deceleration of BmN cells in the G0/G1 phase. Flow cytometry analysis of the cell cycle distribution in PI-stained Per-KD and WT cells found that the Per-KD cell cycle decelerated and the percentage of cells in the G0/G1 phase increased with the time in culture (Figure 4A). The percentage of cells in the G0/G1 phase was higher in the Per-KD group than in the WT group at 24, 48, 72, and 120 h after synchronization (Figure 4B). The percentage of cells in S phase was lower in Per-KD than in WT cells at each assay time (*p* < 0.001, Figure 4C). The percentage in G2/M was higher in Per-KD than in WT cells at 24 and 48 h after synchronization (*p* < 0.001); but then decreased, and at 120 h the percentage in G2/M was lower in Per-KD than in WT cells (*p* < 0.01, Figure 4D). The cell cycle distribution data showed that WT cells cultured for 24–120 h after cell cycle synchronization were mainly distributed in the S phase, and cells in G0/G1 phase gradually decreased in number. The Per-KD cells in S phase increase as those in G2/M decrease. (Figure 4E).

Flow cytometry of PI-labeled cells showed that the proliferation index of Per-KD cells was lower than that of WT cells at 24 (*p* < 0.001) and 120 h (*p* < 0.01) after cell synchronization. The proliferation index remained low in Per-KD cells and did not increase with prolonged culture time as it did in WT cells (Figure 4F). RT-qPCR assays of the transcription of five cell cycle related genes shown in Supplementary Figure S2 are consistent with the flow cytometry results indicative of a blocked cell cycle shown in Figure 4E. Knockdown of *BmPer* gene expression was shown to result in slowed BmN cell proliferation, which is consistent with the observations shown in Figure 2, 3.

### The Process of PCD Is Accelerated in Per-KD Cells

**FIGURE 5:**
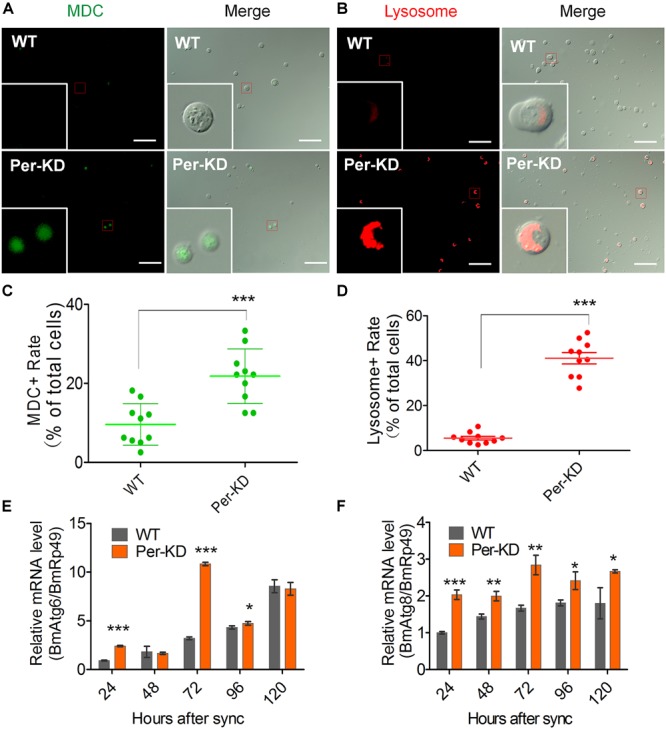
Autophagy was induced by *BmPer* gene knockdown. Autophagy was assayed in WT and Per-KD cells at 24, 48, 72, 96, and 120 h after synchronization. Cell dyeing were performed at 120 h after the synchronization process. **(A)** Monodansylcadaverine (MDC) staining is visible as green fluorescence in autophagosomes and **(B)** Lyso-Tracker Red fluorescence staining shows lysosomes in autophagic cells at 120 h. **(C)** Percentage of positive MDC staining. **(D)** Percentage of positive lysosome staining. **(E,F)** qRT-PCR assay of relative *BmAtg6* **(E)** and *BmAtg8* gene transcription **(F)** after synchronization. Bar = 100 μm. ^∗^*p* < 0.05, ^∗∗^*p* < 0.01, ^∗∗∗^*p* < 0.001, (*n* = 3).

These results suggest that knockdown of *BmPer* expression slowed cell proliferation by slowing the cell cycle, but that accelerated cell death led to a decrease in cell number. Therefore, we investigated changes in the process of PCD in WT and Per-KD cells. Autophagy assay by MDC and Lyso-Tracker Red staining showed that more Per-KD than WT cells included green MDC or red Lyso-Tracker autophagosomes 120 h after developmental synchronization (Figure 5A,C), indicating a significant increase in the autophagy of Per-KD compared with WT cells (*p* < 0.001, Figure 5B,D). Assays of the transcription of autophagy associated genes 6 and 8 (*BmAtg6* and *BmAtg8*) revealed that both were upregulated in Per-KD compared with WT cells at 24, 72, and 96 h (Figure 5E,F).

**FIGURE 6:**
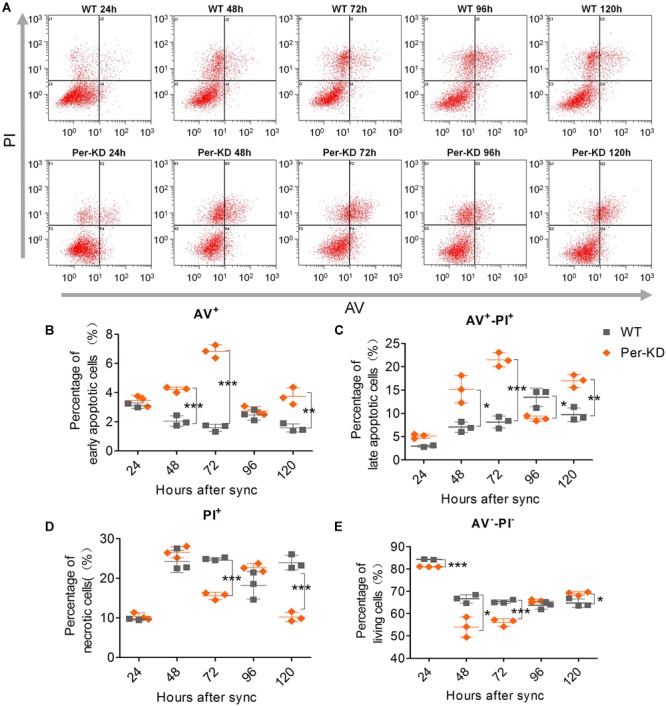
Cell apoptosis and cytoclasis assayed by Annexin V-FITC/PI-FCM. Apoptosis and necrosis were assayed in WT and Per-KD cells every 24 h after synchronization. **(A)** Annexin V-FITC/PI flow cytometry assay of apoptotic cells. **(B)** Annexin V-FITC single-positive apoptotic cells. **(C)** Double-positive Annexin V-FITC/PI positive nonviable apoptotic cells. **(D)** PI single-positive necrotic cells. **(E)** Double-negative Annexin V-FITC living cells. ^∗^*p* < 0.05, ^∗∗^*p* < 0.01, ^∗∗∗^*p* < 0.001, (*n* = 3).

Apoptosis and necrosis were assayed by annexin V-FITC and PI flow cytometry (Figure 6A). The results of the annexin V-FITC single-positive cell assay showed increased early apoptosis of Per-KD compared with WT cells at 48 (*p* < 0.001), 72 (*p* < 0.001), and 120 h (*p* < 0.01). The results at 24 and 96 h were not significantly different (Figure 6B). The PI and annexin V-FITC double-positive assay showed that apoptosis was increased in Per-KD cells at 24, 48 (*p* < 0.05), 72 (*p* < 0.001), and 120 h (*p* < 0.01), but the opposite result (*p* < 0.05) was observed at 96 h (*p* < 0.05, Figure 6C). The results of the PI single-marker assay of cell found that necrosis was lower in Per-KD than in WT cells at 72 and 120 h (both *p* < 0.001). No significant difference was observed at other times (Figure 6D).

Flow cytometry found that the percentage of viable Per-KD cells was lower than that of WT cells at 24 and 72 h (both *p* < 0.001), but higher after 120 h (*p* < 0.05, Figure 6E). The effects of knockdown of *BmPer* expression on apoptosis and necrosis were greater soon after synchronization (24–72 h) than at later stages (120 h). This is inconsistent with the observations shown in Figure 5 of the effects of slowing the cell cycle on proliferation. Therefore, the decrease in the proportion of viable cells with *Per* gene expression knockdown that occurred shortly after synchronization, resulted from apoptosis rather than necrosis.

**FIGURE 7:**
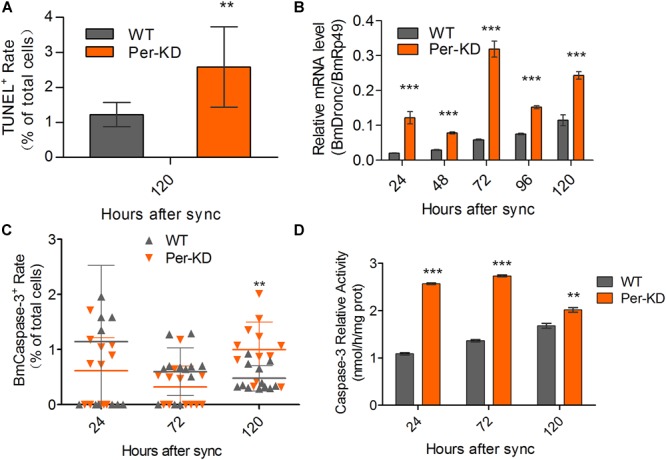
Cell apoptosis induced by *BmPer* gene knockdown. Apoptosis was assayed in WT and Per-KD cells every 24 h after synchronization. **(A)** Percentage of single-positive stained TUNEL cells (*n* = 12). **(B)** *BmDronc* mRNA expression assayed by qRT-PCR. The reference gene was *BmRp49* (*n* = 3). **(C)** BmCaspase-3 immunofluorescence staining (*n* = 12). **(D)** Caspase-3 activity (*n* = 3). Nuclei were visualized by blue DAPI fluorescence. ^∗∗^*p* < 0.01 and ^∗∗∗^*p* < 0.001.

The promotion of apoptosis of BmN cells following knockdown of *BmPer* expression was also assayed by TUNEL staining. Positive TUNEL staining was increased in Per-KD cells compared with WT cells 120 h after synchronization (*p* < 0.01, Figure 7A and Supplementary Figure S3A). qRT-PCR showed that the expression of *BmDronc* mRNA, an apoptosis marker, was higher in Per-KD than in WT cells in the 24–120 h after synchronization (Figure 7B). An immunofluorescence assay found no significant differences in BmCaspase-3 protein expression in Per-KD and WT cells at 24 and 72 h after cell synchronization, but found significantly increased expression in Per-KD cells at 120 h (*p* < 0.01, Figure 7C and Supplementary Figure S3B). BmCaspase-3 activity was significantly higher in Per-KD cells than in WT cells (*p* < 0.01) in the 24–120 h after cell synchronization (Figure 7D). The results indicate that the knockdown of *BmPer* expression induced apoptosis by the caspase pathway.

## Discussion

### A Possible Mechanism of Inhibition of Cell Growth and Proliferation by Knockdown of *BmPer* Gene Expression

The core components of circadian clock signal input negative feedback loop can promote or inhibit cell growth and proliferation (Filipski et al., 2002, 2004; EI Cheikh et al., 2014; Blakeman et al., 2016; Lu et al., 2016; Tao et al., 2017). The mechanisms have not been described. In mammals with multiple *Per* subtypes, the temporal and spatial expression of homologous genes, and the activities of their protein products, are very different (Shearman et al., 2000; Bae et al., 2001; Cermakian et al., 2001; Zheng et al., 2001). Regulation of cell proliferation by the circadian clock system is not conducted via a single pathway.

**FIGURE 8:**
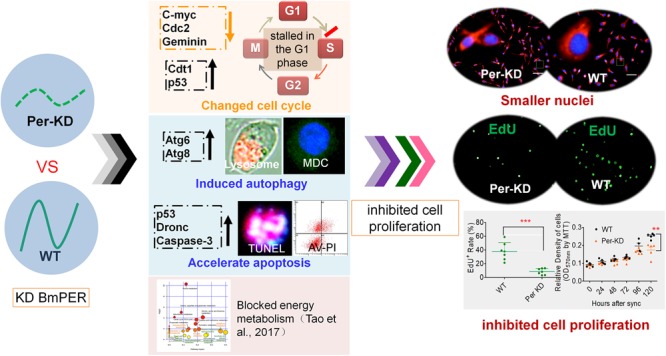
A proposed mechanism of inhibition of the cell cycle associated with knockdown of *BmPer* expression. Knockdown of the *BmPer* gene causes cell cycle deceleration, increased autophagy and apoptosis, or other previously reported changes in cellular metabolism (Tao et al., 2017), ultimately lead to cell shrinkage and inhibition of cell proliferation.

This study evaluated the effects of *Per* knocking-down on cell proliferation using a cell model of a *Period* gene disorder, Per-KD, which was constructed previously with a silkworm ovary cell line (Tao et al., 2017). Comparison of the regulation of cell proliferation and PCD between mutant Per-KD and WT BmN cells revealed that *BmPer* knockdown led to shrinkage of BmN cell nuclei, decelerated cell cycling, increased autophagy and apoptosis, and decreased cell proliferation. The proposed mechanism is shown in Figure 8.

The size of the daughter cells produced by cytokinesis may influence cell cycle progression, with large daughter cells accelerating G1 and G2 and promoting cell cycle progression; small daughter cells have the opposite effects (Barnum and O’Connell, 2014). In this study, the Per-KD cells had smaller nuclei, decreased nuclear DNA synthesis in dividing cells, and a delayed cell cycle with cell development blocked in G0/G1. The observed effects of *BmPer* gene knockdown increased with prolonged culture after synchronization.

Previous studies have shown that a defective circadian clock system can disrupt the cell cycle and affect apoptosis in addition to influencing cell metabolism (Blakeman et al., 2016). *BmPer* knockdown in the silkworm has been reported to suppress cellular glucose metabolism and was correlated with changes in amino acid metabolism and inhibition of cell growth (Tao et al., 2017). Inhibition of cell proliferation has been associated with accelerated apoptosis with impairment of the circadian clock system by knockdown of *mClk* expression in mESCs (Lu et al., 2016). In this study, the observed effects on the regulation of PCD in Per-KD BmN cells included induction of autophagy by the lysosomal pathway and apoptosis by the caspase pathway.

### Effects of the *Period* Gene on the Proliferation Differ in Mammalian and Silkworm Cells

The effects of changes of *Per* gene expression on cell proliferation have not been reported in animal models or normal cells, but a positive correlation of *m/hPer1* and *m/hPer2* gene expression with promotion of cell proliferation and reduction of cell apoptosis has been shown in mammalian tumor models. Mice lacking *mPer2* expression develop lymphoma (Fu et al., 2002), and *hPer2* expression is downregulated in human lymphoma and myeloid malignancy cells (Gery et al., 2005). Downregulation of *hPer1* expression in mouse mammary carcinoma cells or human oral squamous cell carcinoma can alter cell cycle distribution, promote cell cycling, and reduce apoptosis (Borgs et al., 2009; Yang et al., 2009; Soták et al., 2014; Zhao et al., 2016). Downregulation of *Per2* expression in mammals promotes expression of the cell proliferation-associated proteins Cyclin A, Cyclin D1, Mdm2, C-MYC, β-Catenin and Cyclin E, thereby promoting cell proliferation (Fu et al., 2002; Rosbash and Takahashi, 2002; Lee, 2006; Yang and Stockwell, 2008).

In addition, hPER1 is known to be an anti-apoptosis factor in human pancreatic, liver, and gingival cancer (Sato et al., 2009; Sato et al., 2011). Overexpression of *hPer1* inhibits cell proliferation (Gery et al., 2006, 2007) and reduces the expression of Cyclin B1 and WEE1 proteins, promotes apoptosis, and inhibits cell proliferation in HCT116 human colonic cancer cells and in human prostate cancer cells (Gery et al., 2006; Cao et al., 2009). Overexpression of human *hPer2* has been shown to inhibit the growth of the K562 and U937 myeloid leukemia cell, leading to K562 cell proliferation arrest, loss of clonality, and apoptosis (Gery et al., 2005). Overexpression of the mouse *mPer2* gene has been shown to inhibit both tumor formation, and cell proliferation by decreasing Cyclin B1 and Cyclin A2 expression, while promoting *p53* transcription to accelerate apoptosis (Hua et al., 2006; Lee, 2006; Hua et al., 2007; Wood et al., 2008; Yang and Stockwell, 2008). In this study, knockdown of *BmPer* gene expression in BmN cells induced autophagy and apoptosis, and inhibited cell proliferation. This is opposite to the effects of inhibition of *m/hPer1* and *m/hPer2* expression in mammalian cells.

It has been reported that the circadian clock and cell cycle are coordinated via MYC, WEE1, P16, P53 and other coupling factors (Shostak, 2017), the loss of *mPer2* function results in the increased expression of *c-Myc* and partially impairs *p53*-mediated apoptosis (Fu et al., 2002). The expression products of the cellular-oncogene *c-Myc* and cell division cycle 2 gene (*Cdc2*) directly regulated the proliferation of cultured cells, and that C-MYC protein accelerated the progression of cells through G1 and S (Herrera-Merchan et al., 2010). *Cdc2* was shown to regulate cell differentiation from G2 to M (Morgan, 1995). The transcription level of the *Geminin* and Cdc10-dependent transcript 1 gene (*Cdt1*) oscillates throughout the cell cycle and influences nuclear replication and cell division. *Geminin* is not transcribed during the G1 phase and is significantly upregulated during the S, G2, and M phases (Suchyta et al., 2015). *Cdt1* transcription is low in the S phase and high in G1 (Nishitani et al., 2001). The tumor suppressor gene *p53* is a cell cycle suppressor and an important regulator of G1/S checkpoints (Fu et al., 2016). Activation of *p53* gene expression induces cell cycle arrest, promotes apoptosis, and transcription of senescence genes in response to stress, such as DNA damage (Herrera-Merchan et al., 2010). It can thus be seen that the *mPer* gene regulates the cell cycle by its effects on C-MYC, CDC2, P53, and other coupling factors in mammalian cells.

We investigated the transcription of these five genes in a silkworm ovary cell line with *BmPer* expression knockdown (Supplementary Figure S2). Downregulation of *Bmc-Myc, BmCdc2*, and *BmGeminin* at 96 and 120 h after synchronization of cell proliferation, and upregulation of *BmCdt1* and *Bmp53* expression, with decreased cell proliferation and increased apoptosis (Figure 8). It has been reported that knockdown of the *mPer2* gene in mice results in upregulation of *c-Myc* and downregulation of *p53* gene expression as well as promotion of cell proliferation and reduction of cell apoptosis (Fu et al., 2002). These effects differ in mammalian and silkworm cells and can be explained as follows.

(1) Effects of the *BmPer* and *mPer* genes on cell proliferation may depend on species differences in PER protein function in the circadian clock transcription–translation feedback loop (TTFL). However, we believe that *BmPer* or *mPer* knockdown affects cell proliferation and apoptosis of both mice and BmN cells via mediating C-MYC, P53 and other factors. In mammals, the most widely accepted explanation of the TTFL is that mPER independently or together with mCRYs (mCRYs/mPERs) binds CLOCK/BMAL1, with feedback inhibition of *mCry* and *mPer* genes transcription (Reppert and Weaver, 2001; Ko and Takahashi, 2006). On the other hand, some reports indicate that mPER2 can independently promote gene transcription at some time in the TTFL (Akashi et al., 2014; Ye et al., 2014). The available evidence indicates that mPER may have dual functions in different situations. The *Danaus plexippus* and *Bombyx mori* are both members of order Lepidoptera, in which DpCRY2 shuts down CLOCK-CYCLE-mediated transcription. DpPER likely helps to transport DpCRY2 from the cytoplasm to nucleus, but has no inhibitory feedback effect on DpPER (Zhu et al., 2008).

*Bombyx mori* has only a single *Period* gene and protein, but there are multiple *mPer* gene subtypes in mice. It has been reported that mPER1 and mPER2 proteins have distinct and complementary roles in the mouse clock mechanism (Bae et al., 2001; Zheng et al., 2001). The roles of the *mPer2* and *mPer1* genes on cell proliferation and apoptosis have not been investigated after knockout of their complementary genes (*mPer1* or *mPer2*). Consequently, the effects of *mPer1* or *mPer2* on cell proliferation and apoptosis may not reflect the influence of *mPer* genes.

(2) Metabolism affects cell proliferation in complex ways. Previous studies have shown that fast-dividing pluripotent stem cells are usually highly glycolytic, with the energy provided by glycolysis as key driver of cell proliferation (Folmes et al., 2012; Zhang et al., 2012). Mice deficient in *mPer2* have significantly increased circulating insulin levels compared with WT mice, and that can lead to upregulation of glucose metabolism (Zhao et al., 2012). In mouse models, the anterior tibialis muscle in *mPer2* knockout mice has increased levels of glycolytic enzymes such as triose phosphate isomerase and enolase (Bae et al., 2006). However, double knockout of *mPer1* and *mPer2* has the opposite effect on blood insulin levels (Lamia et al., 2008). We previously assayed cell metabolomics by gas chromatography/liquid chromatography-mass spectrometry (GC/LC-MS) and found that knockdown of *BmPer* gene expression resulted in significant inhibition of glycometabolism, the amino acids that used glucose metabolites as a source were also downregulated. The activities of key glycolytic enzymes, such as hexokinase (BmHK), phosphofructokinase (BmPFK), and citrate synthase (BmCS), were significantly decreased and transcription of their encoding genes, as well as that of pyruvate kinase, were significantly downregulated (Tao et al., 2017). The results clearly showed that inhibition of the circadian clock gene *BmPer* repressed glycometabolism. The results differ from the increase in glycometabolism caused by knockout of the *mPer2* gene (Bae et al., 2006; Zhao et al., 2012), but are consistent with changes in the glycometabolism in *mPer1*/*mPer2* double knockout mice (Lamia et al., 2008). Species differences in the activities of gene products may thus help explain the opposite effects of *BmPer* knockdown and *mPer1* or *mPer2* knockdown or deletion on cell proliferation.

## Conclusion

Knockdown of *BmPer* expression in *Bombyx mori* blocked the BmN cell cycle, decreased nuclear size, accelerated apoptosis and autophagy, and inhibited cell proliferation.

## Author Contributions

S-QX and J-FQ conceived this project. XL, J-FQ, W-ZC, X-FL, HT, and Y-HS performed the research. XL, J-FQ, and S-QX analyzed the data and wrote the manuscript. All authors participated in the revision of this manuscript.

## Conflict of Interest Statement

The authors declare that the research was conducted in the absence of any commercial or financial relationships that could be construed as a potential conflict of interest.
