# Supplementary material for: Inhibition of Period Gene Expression Causes Repression of Cell Cycle Progression and Cell Growth in the Bombyx mori Cells
Source: Front Physiol. 2019 May 3;10:537. doi: 10.3389/fphys.2019.00537 (PMC6509393; doi:10.3389/fphys.2019.00537)
Supplement: Supplementary file 1 [file Data_Sheet_1.docx]

# Supplementary Figures and Tables

## Supplementary Table

**Table S1 Primers for qRT-PCR**

| Primer names | Sequnces | GeneID |
| --- | --- | --- |
| *BmAtg6* | GTTATACGGTTCGGGTGG  TGGAGTACGCATGTGGTG | 100216352 |
| *BmAtg8* | AAGGCTAGGCTTGGAGAC  CAGATGTGGGTGGAATGA | 692938 |
| *Bm**Cdc2* | GTACTGTCACCAAAGACGGAT  CACTGGAACACCGAATGCTC | 693060 |
| *Bm**Cdt1* | GGATGATATAGCCACAACCAA  TGTTTTCTTAGCAGCCGTA | 100873154 |
| *Bm**C-myc* | AAATGCAGATCCCCGCCACGA  ACTTCTCCCGCTCGATATCCTCT | 100862782 |
| *Bm**Dronc* | TGTGGCTGTCTTCCTTC  ATCTAAGTCTGTGCCCTC | 100500764 |
| *Bm**Geminin* | GAAACTCAGGCCAATCTCG  TGCCAATATTTCTCTGATGCT | 100873155 |
| *Bm**p53* | ACTTCAGCGTCGAAATCCACA  TTCACGTAGATCCGGTTCAGC | 100384887 |
| *BmRp49* | CAGGCGGTTCAAGGGTCAATAC  TGCTGGGCTCTTTCCACGA | 6100595 |

## Supplementary Figures


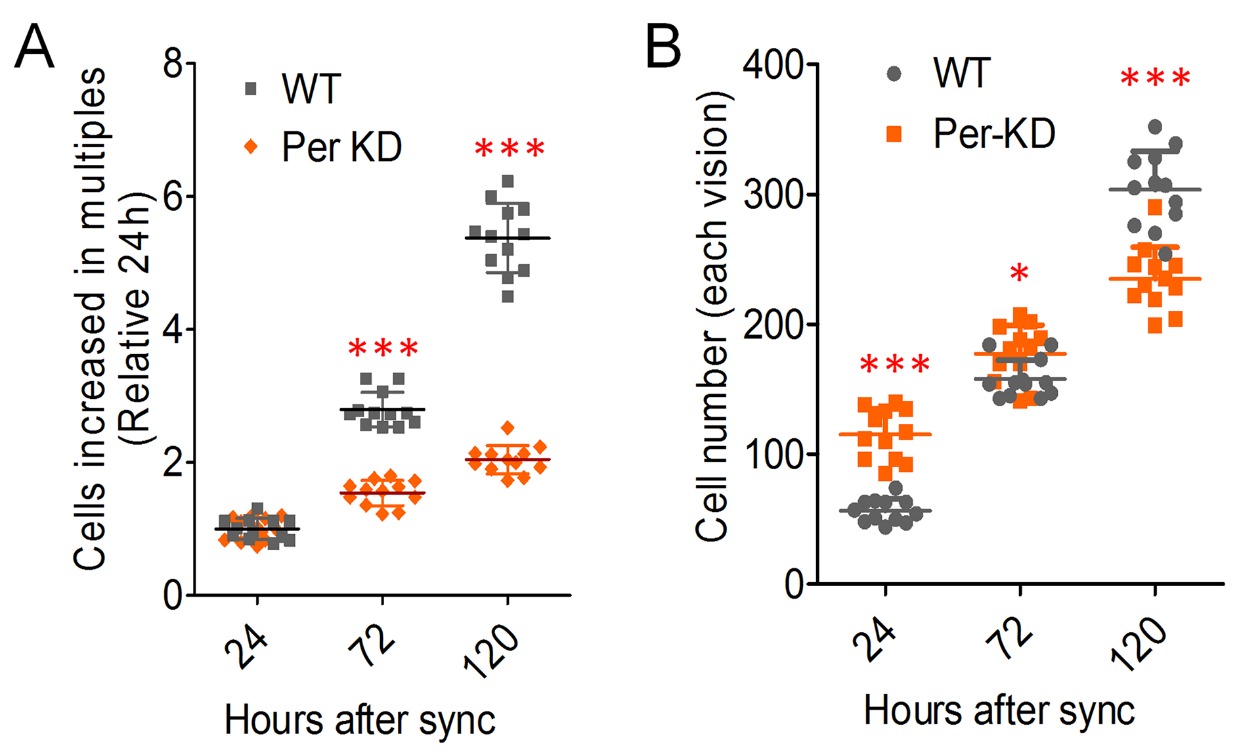


**FIGURE S1 Effect of *BmPer* gene knockdown on cell number approximately in BmN cells.** The 1.5×10^5^ Cells (1 mL, 1.5×10^5^ cells/mL) were transferred to a coverslip when they reached 70% confluence. After the cells were completely adherent (at least 96 h) the cells were synchronized 24 h using serum-free Grace insect medium. Then, replacement media using Grace insect medium added 10% (v/v) FBS. The nuclei were stained with DAPI at 24 h, 72 h and 120 h after replacing normal medium. (A) Cells increased in multiples relative 24 h. (B) Cell nuclei number in each microscopic fields. * p< 0.05, *** p< 0.001 indicate significant differences (n = 3 plates, observations and nuclei number statistics were repeated five microscopic fields). WT, wild-type BmN cells; Per-KD, *BmPer* knockdown BmN cells.

**
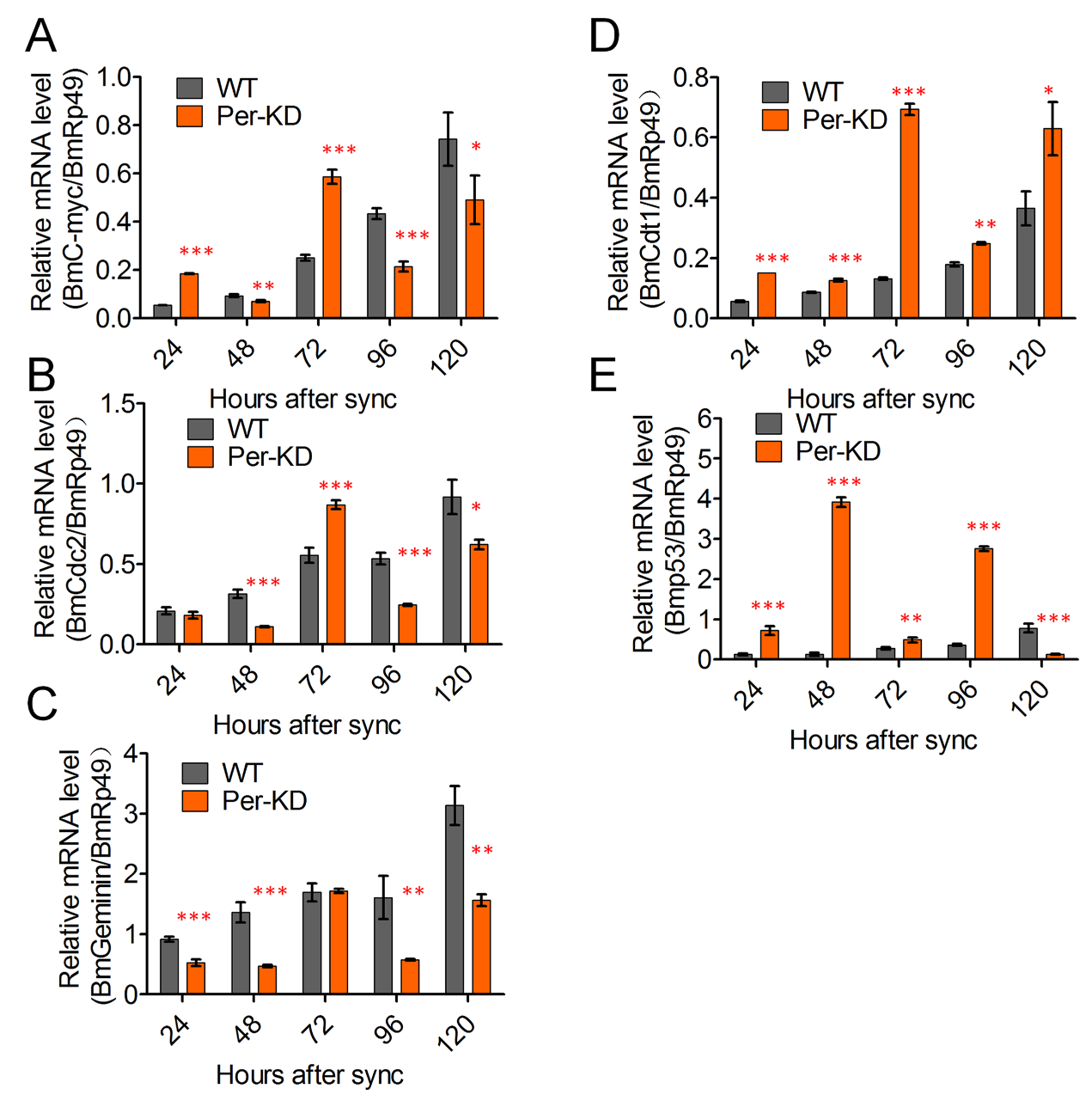
**

**FIGURE S2 The cell cycle related genes expression changed in Per-KD cells.** The 1.5×105 Cells (1mL, 1.5×105 cells/mL) were transferred to a coverslip when they reached 70% confluence. After the cells were completely adherent (at least 96 h) the cells were synchronized 24 h using serum-free Grace insect medium. Then, replacement media using Grace insect medium added 10% (v/v) FBS. (A) Cellular-oncogene C-myc (*C-myc*), (B) cell division cycle 2 gene (*Cdc2*), (C) *Geminin*, (D) Cdc10-dependent transcript 1 gene (*Cdt1*) and (E) tumor suppressor gene p53 (*p53*) transcripts were analyzed by qRT-PCR with *BmRp49* as an internal control. WT, wild-type BmN cells; Per-KD, *BmPer* knockdown BmN cells. *p ≤ 0.05 (repeated three times). The marks of *, **and *** mean the difference between the *BmPer* cells and its WT reached the significant level of p<0.05, p< 0.01, p< 0.001, respectively (n=3).
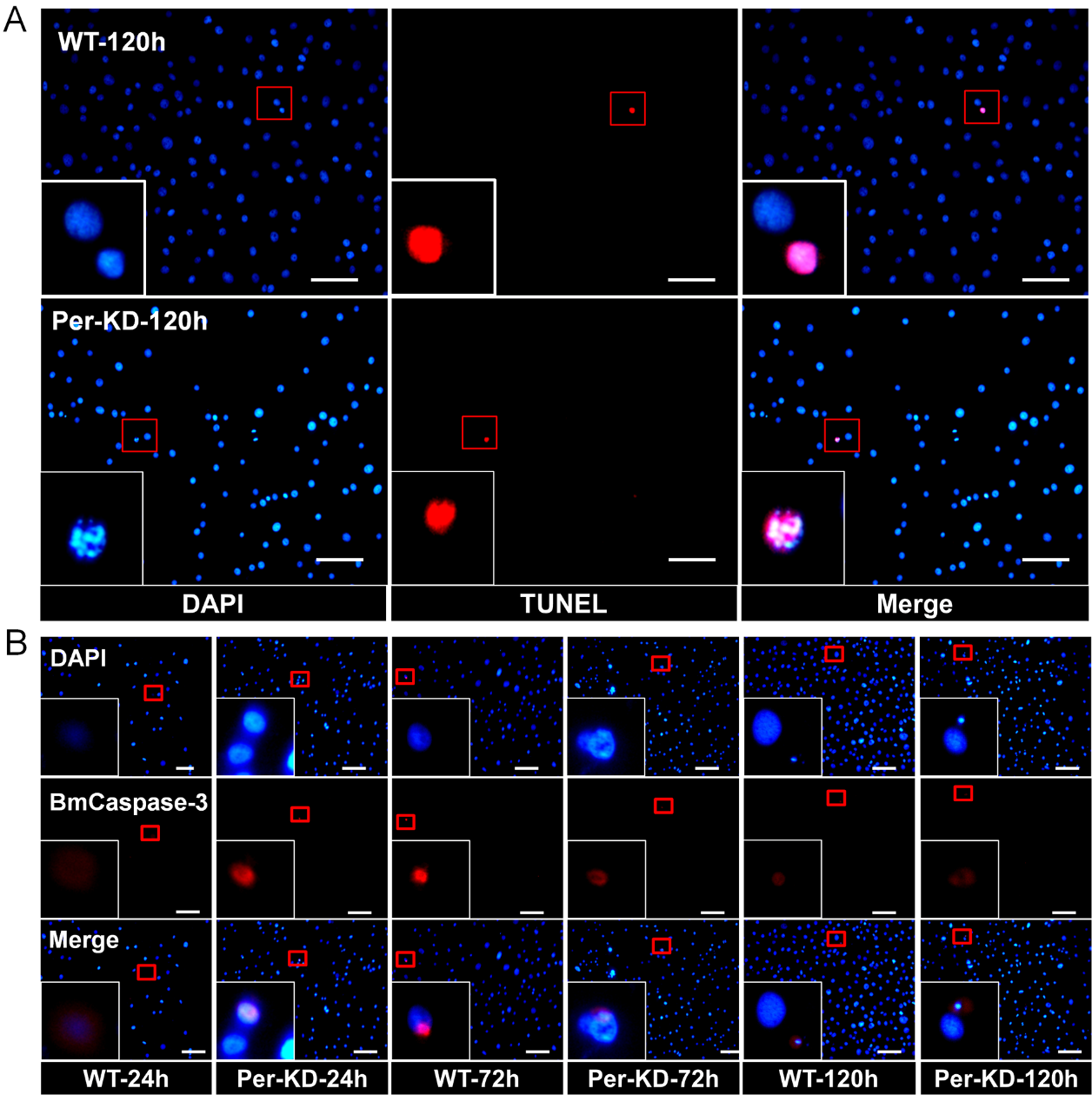


**FIGURE S3 (A) TUNEL and (B) immunofluorescence detection.** The required number of WT and Per-KD cells were detected at 24 h, 72 h and 120 h after synchronization. Bar =100μm. In picture A, the red fluorescence show the apoptotic cell nucleus by TUNEL staining. In picture B, the red fluorescence show the BmCaspase-3 by immunofluorescence. Cell nuclei were blue stained with 4'-6-diamidino-2-phenylindole (DAPI). Refer to Li et al. (2017), the cell sample (20 μL) was mixed with 180 μL of HBSS, allowed to stand, fixed, and washed with PBS, and then 1 mL of cell permeabilization solution was added to it. The slide was allowed to stand for 60 min at room temperature and was then washed with PBS. 600 μL Anti-caspase-3 antibody (1:200, 9661s, CST, USA) was added dropwise and the slide was incubated in the dark overnight at 4°C with shaking. After the slide was washed three times with PBS, 600 μL of secondary antibody (Alexa Fluor 594-conjugated Goat Anti-Mouse IgG, AS054, ABclonal, Wuhan, China) was added dropwise and incubated at 37°C in the dark for 1 h on a shaker. After the slide was washed with PBS, Antifade Mounting Medium was added dropwise. The characteristic red fluorescence of the secondary antibody was observed.
